# Committee report: Questionnaire survey on the treatment of COVID-19 in patients receiving dialysis therapy

**DOI:** 10.1186/s41100-022-00405-8

**Published:** 2022-04-25

**Authors:** Ayumi Yoshifuji, Munekazu Ryuzaki, Yuki Uehara, Norio Ohmagari, Toru Kawai, Yoshihiko Kanno, Kan Kikuchi, Hiroshi Kon, Ken Sakai, Toshio Shinoda, Yaoko Takano, Junko Tanaka, Kazuhiko Hora, Yasushi Nakazawa, Naoki Hasegawa, Norio Hanafusa, Fumihiko Hinoshita, Keita Morikane, Shu Wakino, Hidetomo Nakamoto, Yoshiaki Takemoto

**Affiliations:** 1 Infection Control Committee, The Japanese Society for Dialysis Therapy, Tokyo, Japan; 2 Department of Nephrology, Tokyo Saiseikai Central Hospital, 1-4-17 Mita, Minato-ku, Tokyo, 108-0073 Japan

**Keywords:** Coronavirus disease 2019, Renal replacement therapy, Steroids, Dialysis

## Abstract

**Background:**

Patients with coronavirus disease 2019 (COVID-19) who receive dialysis therapy develop more severe disease and have a poorer prognosis than patients who do not. Although various data on the treatment of patients not receiving dialysis therapy have been reported, clinical practice for patients on dialysis is challenging as data is limited. The Infection Control Committee of the Japanese Society for Dialysis Therapy decided to clarify the status of treatment in COVID-19 patients on dialysis.

**Methods:**

A questionnaire survey of 105 centers that had treated at least five COVID-19 patients on dialysis was conducted in August 2021.

**Results:**

Sixty-six centers (62.9%) responded to the questionnaire. Antivirals were administered in 27.7% of facilities treating mild disease (most patients received favipiravir) and 66.7% of facilities treating moderate disease (most patients with moderate or more severe conditions received remdesivir). Whether and how remdesivir is administered varies between centers. Steroids were initiated most frequently in moderate II disease (50.8%), while 43.1% of the facilities initiated steroids in mild or moderate I disease. The type of steroid, dose, and the duration of administration were generally consistent, with most facilities administering dexamethasone 6 mg orally or 6.6 mg intravenously for 10 days. Steroid pulse therapy was administered in 48.5% of the facilities, and tocilizumab was administered in 25.8% of the facilities, mainly to patients on ventilators or equivalent medications, or to the cases of exacerbations. Furthermore, some facilities used a polymethylmethacrylate membrane during dialysis, nafamostat as an anticoagulant, and continuous hemodiafiltration in severe cases. There was limited experience of polymyxin B-immobilized fiber column-direct hemoperfusion and extracorporeal membrane oxygenation. The discharge criteria for patients receiving dialysis therapy were longer than those set by the Ministry of Health, Labor and Welfare in 22.7% of the facilities.

**Conclusions:**

Our survey revealed a variety of treatment practices in each facility. Further evidence and innovations are required to improve the prognosis of patients with COVID-19 receiving dialysis therapy.

## Background

Coronavirus disease 2019 (COVID-19) caused by severe acute respiratory syndrome coronavirus 2 (SARS-CoV-2) was first reported in China in December 2019 and resulted in a global pandemic that has not yet been controlled. As of December 28, 2021, more than 278 million people worldwide were affected by COVID-19, and more than 5.4 million people had died [1]. In Japan, by December 29, 2021, 1.73 million people were affected by COVID-19 with more than 18,000 deaths [2]. Dialysis patients are known to be at a risk for severe disease [3]. The prognosis of COVID-19 patients with chronic kidney disease is poor, especially in those undergoing hemodialysis, in whom the mortality rate ranges from 14% [4] up to 50% in severe COVID-19 cases [5]. In Japan, as of December 23, 2021, 2,676 dialysis patients were affected by COVID-19, with 423 deaths and a mortality rate of 15.8% [6]. Although a treatment guide for COVID-19 has been published in Japan [7], it is basically based on evidence from non-dialysis patients. In addition, some of the drugs used to treat COVID-19 patients have not been shown to have an adequate safety profile in dialysis patients, resulting in difficulties in making treatment decisions. To summarize the treatment methods used by each institution and to improve the prognosis of dialysis patients, a questionnaire survey was conducted to evaluate the treatment provided by each facility in Japan to allow further refinements in the future.

## Methods

In August 2021, a questionnaire on the treatment of COVID-19 in patients receiving dialysis therapy was distributed to 105 facilities that was registered with the Japanese Association of Dialysis Physicians or the Japanese Society for Dialysis Therapy (JSDT), and had accepted at least five COVID-19 patients who were receiving dialysis therapy. They were requested to respond anonymously. Disease severity was determined in accordance with the guidelines for the treatment of COVID-19 published by the Ministry of Health, Labor and Welfare (MHLW) [7]. Mild disease was defined as oxygen saturation (SpO_2_) ≥ 96% and no respiratory symptoms or only cough without dyspnea; moderate disease I was defined as 93% < SpO_2_ < 96% and findings of dyspnea or pneumonia; moderate disease II was defined as SpO_2_ ≤ 93% and the need for oxygen therapy; and severe disease was defined as admission to the intensive care unit (ICU) or the need for a ventilator.

Details of the questionnaire are presented in Table 1. Briefly, questionnaire included about (1) presence/absence and type of antivirals, (2) presence/absence and type of steroids, (3) the use of steroid pulse therapy and tocilizumab, (4) the choice of dialysis membrane and anticoagulants, (5) the use of Polymyxin B-immobilized fiber column-direct hemoperfusion (PMX-DHP) and Continuous hemodiafiltration (CHDF), and (6) Do Not Attempt Resuscitation (DNAR). Along with optional questions, open-ended questions were included to collect valid and honest opinions. Then, each question in the questionnaire was collated. This survey was proposed by the Infection Control Committee of JSDT and was approved by the Board of Directors of JSDT as an urgent matter.

**Table 1:** Questionnaire on the treatment of COVID-19 in patients receiving dialysis therapy

General questions
Select the severity of the admitted patients (mild, moderate disease I; moderate disease II; severe).
Describe the number of dialysis patients (including peritoneal dialysis and combination therapy) with COVID-19.
(1) Were antivirals administered to patients with mild disease?
Describe the type of antivirals administered.
(2) Were antivirals were administered to patients with moderate I or more severe disease?
Describe the type of antivirals administered.
Describe the protocol of remdesivir if administered.
(3) When were steroids initiated? Select the severity of the disease (mild, moderate disease I; moderate disease II; severe).
(4) Describe the type, dose, method, and duration of steroids (the standard therapy consists of dexamethasone 6 mg for 10 days).
(5) Was steroid pulse therapy ever applied?
Describe the treatment protocols and their criteria.
(6) Was the duration of steroid administration prolonged?
What parameters were used when extending the duration of treatment?
(7) Was tocilizumab or baricitinib ever applied?
Describe the criteria.
(8) Were the membranes and anticoagulants changed during dialysis?
Specify the details.
(9) Was continuous hemodiafiltration (CHDF) or polymyxin B-immobilized fiber column-direct hemoperfusion used in severe cases?
Describe the criteria for starting CHDF, membranes, and anticoagulants.
Describe the criteria for using PMX-DHP.
(10) Have you ever applied extracorporeal membrane oxygenation?
Describe the criteria and its prognosis if you have.
Describe the reasons if you have not.
(11) Are the discharge criteria for patients undergoing dialysis the same as those for general patients?
Describe the discharge criteria.
(12) Do you explain `Do Not Attempt Resuscitation' on admission?

## Results

**Fig. 1:**
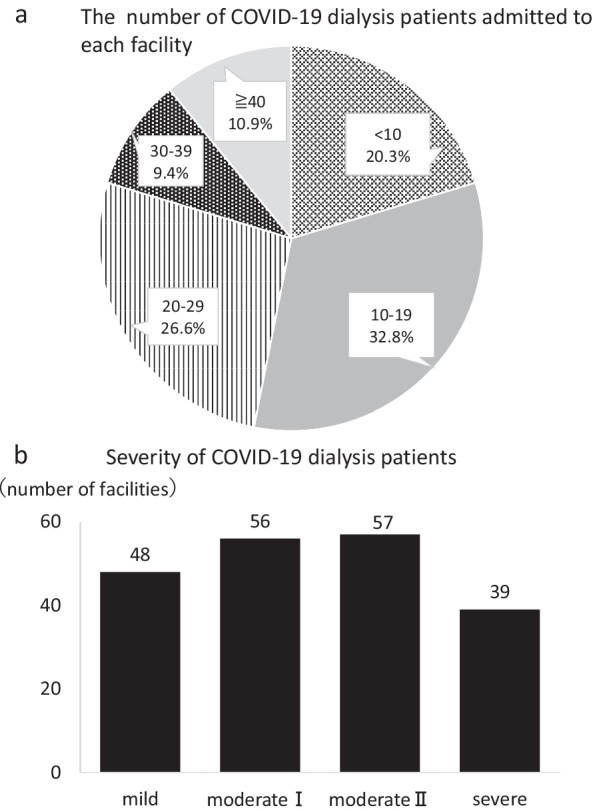
The profiles of facilities admitting COVID-19 patients receiving dialysis therapy. **a** The facilities were divided into five groups by the number of COVID-19 dialysis patients admitted to each facility; < 10 patients, 10–19 patients, 20–29 patients, 30–39 patients, and ≥ 40 patients. **b** Severity of the COVID-19 dialysis patients that were admitted. The severity at each facility accepted for hospitalization was divided into mild, moderate I, moderate II, and severe, which accounted for 48, 56, 57, and 39 facilities, respectively

**Fig. 2:**
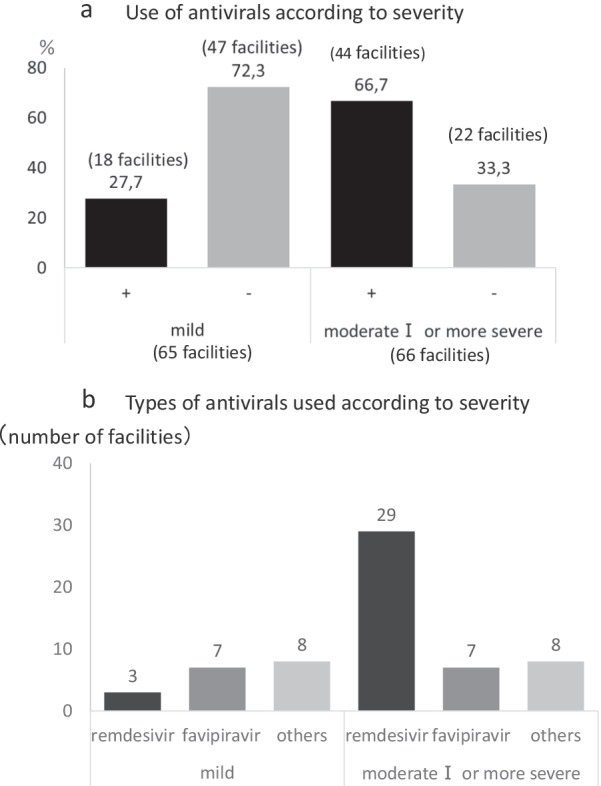
The details in antivirals. **a** The use of antivirals was evaluated according to severity. A total of 66 facilities responded the survey (65 facilities for mild and 66 facilities for moderate I or more severe). “ + ” indicates administration of the antivirals, and “-” indicates no administration of the antivirals. Among those with mild disease, 27.7% of facilities (18 facilities) administered antivirals, and 72.3% (47 facilities) did not. In patients with moderate disease I or more severe conditions, 66.7% (44 facilities) administered antivirals, and 33.3% (22 facilities) were not. **b** The numbers of facilities treated with either remdesivir or favipiravir at each severity were evaluated. Among 18 facilities administering antivirals in mild disease, 3 facilities (16.7%) administered remdesivir, 7 facilities (38.9%) administered favipiravir and other 8 facilities (44.4%) did not answer the question or administered anti-SARS-CoV-2 monoclonal antibodies. Among 44 facilities administering antivirals in moderate disease I or more severe disease, 29 facilities (65.9%) administered remdesivir, 7 facilities (15.9%) administered favipiravir and other 8 facilities (18.2%) did not answer the question or administered anti-SARS-CoV-2 monoclonal antibodies

A questionnaire survey was distributed to 105 facilities that had accepted five or more dialysis patients with COVID-19, of which 66 facilities (62.9%) responded. Figure 1a shows the number of COVID-19 patients admitted to the facilities who responded to the questionnaire up to August 2021. Figure 1b shows the severity at each facility accepted for hospitalization (including duplicates). The use of antiviral drugs was evaluated according to severity. Among those with mild disease, 27.7% of facilities (18 out of 65 facilities) administered antivirals (Fig. 2a). 16.7% of the facilities (3 out of 18 facilities) administered remdesivir, and 38.9% of them (7 out of 18 facilities) administered favipiravir (Fig. 2b). In patients with moderate disease I or higher, 66.7% of facilities (44 out of 66 facilities) administered antivirals (Fig. 2a). 65.9% of the facilities (29 out of 44 facilities) administered remdesivir and 15.9% of them (7 out of 44 facilities) administered favipiravir (Fig. 2b). The administration protocols of remdesivir were (1) 200 mg on the first day and 100 mg on the second and subsequent days (usual dose) in 55.2% (16 out of 29 facilities), (2) 100 mg 4 h before dialysis (only on dialysis days) in 27.6% (8 out of 29 facilities), and others in 17.2% (5 out of 29 facilities) (Table 2).

**Table 2:** The protocols of remdesivir (29 facilities responded)

Methods	Duration	Number (%)**
Administration by the normal dose*	5 days	6 (20.7)
	6–9 days	6 (20.7)
	10 days	2 (6.9)
	Not shown	2 (6.9)
Administration 4 h before dialysis	5–6 times	4 (13.8)
	Not shown	4 (13.8)
Others		5 (17.2)

^*^Usual daily dose: day 1, 200 mg; day 2 onwards, 100 mg

^**^Percentage for the facilities providing remdesivir protocol (29 facilities)

Other dosage methods

-100 mg three times after dialysis

-100 mg on consecutive days (specific duration is not shown)

-100 mg (day 1), 50 mg (days 2–5)

-200 mg (day 1), 100 mg (days 2 and 3), dialysis thereafter for a total of 10 vials

-200 mg (day 1), 100 mg (days 2 and 3), dialysis thereafter

Perform 3 h of CHDF 4 h after administration

**Fig. 3:**
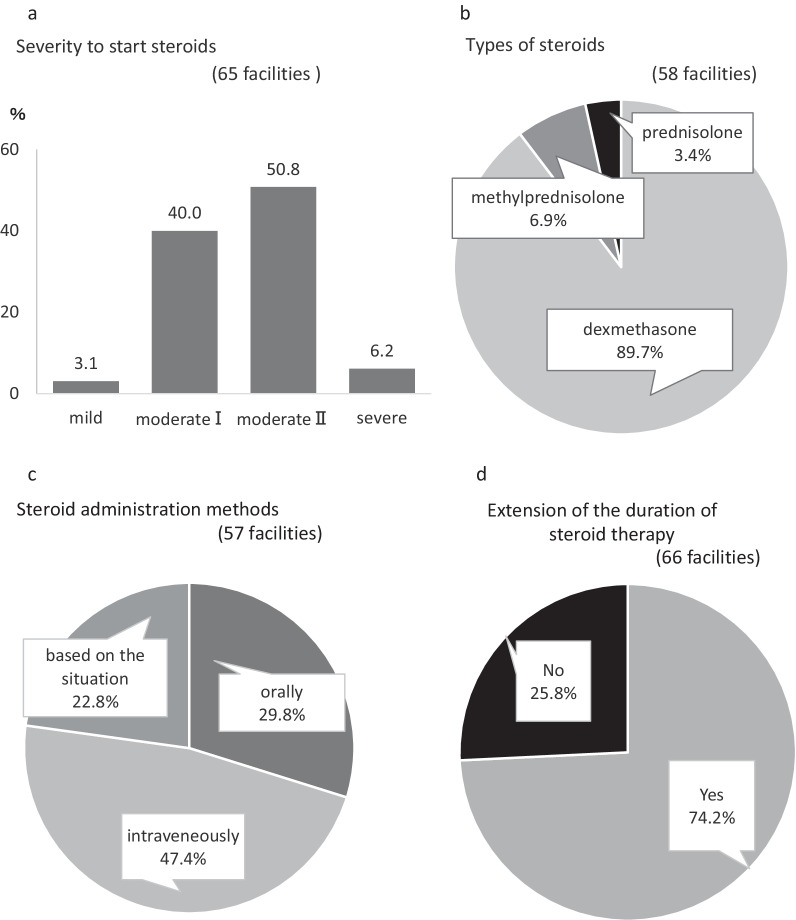
The details in steroid therapy. **a** The severity at the time of steroid treatment initiation was evaluated. A total of 65 facilities responded to the survey. Two facilities (3.1%) were classified as mild, 26 (40.0%) as moderate I, 33 (50.8%) as moderate II, and 4 (6.2%) as severe. **b** The types of steroids used were also evaluated. A total of 58 facilities responded to the survey: dexamethasone was used at 52 facilities (89.7%), methylprednisolone was used at 4 facilities (6.9%), and prednisolone was administered at 2 facilities (3.4%). **c** The method of steroid administration was evaluated. A total of 57 facilities responded: 17 (29.8%) administered steroids orally, 27 (47.4%) administered steroids intravenously, and 13 (22.8%) decided the route according to the situation. **d** The duration of steroid use was also evaluated. A total of 66 facilities responded to the survey: 49 (74.2%) extended duration of steroid use

When we queried when to initiate steroid therapy in terms of the disease severity, 3.1% of the facilities started in mild condition, 40.0% in moderate I, 50.8% in moderate II, and 6.2% in severe (Fig. 3a). Regarding the type of steroids used, dexamethasone was the most commonly used steroids, followed by methylprednisolone, and then prednisolone (Fig. 3b). All the facilities using dexamethasone used at doses of 6 mg orally (6.6 mg intravenously) or 0.15 mg/kg; the methylprednisolone was used at a dose of 1 to 2 mg/kg. Regarding the method of steroid administration, 29.8% administered steroids orally, 47.4% administered steroids intravenously, and 22.8% decided the route according to the situation (Fig. 3c). Regarding extension of the duration of steroids (against a standard 10-day course), 74.2% reported extending the duration of steroids (Fig. 3d). Indicators of prolonged steroid use included respiratory status (oxygen demand) and imaging findings of chest radiography and chest computed tomography (Table 3). Moreover, when queried regarding steroid pulse therapy, 48.5% (32 out of 66 facilities) reported using this therapy. The dosage of steroid pulse therapy is shown in Table 4. The criteria for the use of steroid pulse therapy included ventilator or equivalent status, exacerbation of symptoms, and failure to respond to conventional therapy (Table 5). When queried regarding the use of tocilizumab and baricitinib, 25.8% (17 out of 66 facilities) reported using tocilizumab, and 3.0% (2 out of 66 facilities) reported using baricitinib. Tocilizumab was also used more frequently in patients on ventilators or equivalent medications and during exacerbations (Table 6).

**Table 3:** Indicators for determination of steroid extension (49 facilities responded)

Indicators "※Multiple answers	Number (%)
Respiratory condition (oxygen demand)	35 (71.4)
X-ray or computed tomography findings	16 (32.7%)
Body temperature	9 (18.4%)
Inflammatory markers	9 (18.4%)
General conditions	2 (4.1%)

**Table 4:** Dose of steroid pulse therapy (21 facilities responded)

Dose (methylprednisolone)	Number (%)
1000 mg	6 (28.6)
500–1000 mg	7 (33.3%)
500 mg	4 (19.0%)
250 mg	3 (14.3%)
125–250 mg	1 (4.8%)

**Table 5:** Criteria for steroid pulse therapy (32 facilities responded)

Criteria	Number (%)
On ventilator or equivalent	8 (25.0%)
Exacerbation	5 (15.6%)
No response to conventional treatment	4 (12.5%)
Oxygen demand	2 (6.3%)
Not shown	13 (40.6%)

Furthermore, to evaluate the possibility of switching dialysis membranes and anticoagulants during dialysis, 18.2% (12 out of 66 facilities) responded to switch. 9.1% (6 out of 66 facilities) changed to nafamostat, 3.0% (2 out of 66 facilities) changed to polymethylmethacrylate (PMMA), and 3.0% (2 out of 66 facilities) increased the membrane surface area to reduce the duration of dialysis (Table 7). Regarding the use of CHDF and PMX-DHP in severe cases, 46.3% (25 out of 54 facilities) responded to use CHDF, and 3.7% (2 out of 54 facilities) used PMX-DHP. Twelve percent (3 out of 25 facilities) used nafamostat as an anticoagulant, and 16.0% (4 out of 25 facilities) used an Polyethyleneimine-coated polyacrylonitrile (AN69ST) membrane (Table 8).

**Table 6:** Criteria for tocilizumab (17 facilities responded)

Criteria	Number (%)
On ventilator or equivalent	4 (23.5)
Exacerbation	4 (23.5)
No response to conventional treatment	1 (5.9)
Pneumoniae without Tb and HBV	1 (5.9)
Not shown	3 (17.6)

**Table 7:** The alterations of methods for anticoagulants or membrane (66 facilities responded)

Methods	Number (%)
Nafamostat	6 (9.1)
PMMA membrane	2 (3.0)
Increase in membrane area	2 (3.0)

**Table 8:** Strategies in the use of CHDF (25 facilities responded)

Strategies	Number (%)
Nafamostat	3 (12.0)
AN69ST membrane	4 (16.0)
PMMA membrane	1 (4.0)

Ten facilities among responded 66 facilities (15.2%) reported having experience with extracorporeal membrane oxygenation (ECMO) in patients undergoing dialysis. The reasons for the avoidance of using ECMO were a lack of equipment and a lack of appropriate patients (Table 9).

**Table 9:** Reasons for non-enforcement of ECMO (56 facilities responded)

Reason	Number (%)
No facilities	20 (35.7)
No applicable cases	8 (14.3)
No application for patients undergoing dialysis	3 (5.4)
Lack of staff	2 (3.6)

Regarding discharge criteria, 22.7% (15 out of 66 facilities) extended the length of hospital stay for dialysis patients compared to the general population. The reasons included a longer time to confirm negative polymerase chain reaction (PCR) as well as antigen tests and the immunocompromised condition of the patients (Table 10). Finally, when queried about discussing Do Not Attempt Resuscitation (DNAR) on admission, 56.1% (37 out of 66 facilities) reported explaining it to all patients, 40.9% (27 facilities) reported explaining it to a limited number of patients, and 3.0% (2 facilities) reported not explaining it at all.

**Table 10:** Reasons for discharge criteria extension (15 facilities responded)

Reason	Number (%)
Negative PCR or antigen test	7 (46.7)
Extension according to own institution's rules	4 (26.7)
Problems with the acceptance facilities	2 (13.3)

## Discussion

Dialysis patients affected by COVID-19 are more severely ill and have a higher mortality rate [3–5]. Therefore, JSDT has recommended all patients on dialysis who are diagnosed as COVID-19, including those with mild disease, to be hospitalized. As there is little evidence to support the use of drugs in patients with dialysis therapy, each institution has made efforts to treat these patients. The Infection Control Committee of JSDT conducted a questionnaire survey to summarize the approaches taken by each facility and encourage future treatment.

The first question was about the appropriate timing to start antivirals. This survey revealed that antivirals were administered 27.7% of the facilities to patients with mild disease (Fig. 2a). Favipiravir was used more frequently than remdesivir in patients with mild disease (Fig. 2b). Although favipiravir has been shown to be effective in patients with mild to moderate disease I in a prospective, randomized, open-label trial [8] and can be used in patients on dialysis with great expectations, large clinical trials have not demonstrated the efficacy of favipiravir, gradually leading to their reduced use. Remdesivir is also not recommended for patients undergoing hemodialysis. Moreover, remdesivir was indicated for patients with moderate or more severe disease until 26th of January 2022, which might have prevented its use. In contrast, in patients with moderate disease I or more severe conditions, the use of favipiravir decreased and the use of remdesivir increased (Fig. 2b). However, in patients with impaired renal function, excretion of sulfobutylether-beta-cyclodextrin, which solubilizes excipient remdesivir, is delayed [9]. Therefore, the use of remdesivir in patients with an estimated glomerular filtration rate of < 30 mL/min/1.73 m^2^ should be considered only when the therapeutic benefit outweighs the risk due to concerns about the hepatic and renal tubular sequelae caused by its accumulation. There are no fixed dosing regimens, and the dosing methods are variable, as shown in Table 2. Aiswarya et al. proposed up to six doses in hemodialysis patients 4 h before the dialysis session [9], and Thakare et al. proposed the usual dose (200 mg initially, then 100 mg/day) [10]. Kikuchi et al. investigated the efficacy of remdesivir in the treatment of COVID-19 in patients receiving dialysis therapy in”a nationwide cohort study”, which showed that remdesivir was effective in shortening the hospital stay and reducing the risk of mortality [11]. These reports led to an increase in the number of centers administering remdesivir; however, many centers were still unable to administer remdesivir due to concerns about its effects and safety, thus remaining used at a low rate in patients with moderate disease I and more severe. Further studies on the administration and safety of remdesivir are expected to ensure its safe use.

Second, severity applied to steroid initiation was assessed. The questionnaire survey revealed that steroids were administered to patients with mild or moderate I disease who did not require oxygen (Fig. 3a). The open-label randomized evaluation of COVID-19 therapy (RECOVERY) study in the UK, which provided the rationale for the use of dexamethasone, showed that steroids significantly reduced mortality in patients with oxygen demand or ventilator management but did not result in a significant difference in patients with no oxygen demand [12]. Furthermore, subsequent reports have shown that steroids are associated with increased mortality in patients with no oxygen demand [13] and are thus not recommended. Therefore, it is crucial to limit steroid administration to patients with oxygen demand. In addition, evidence of steroid administration in patients receiving dialysis therapy is limited. Toçoglu et al. studied the efficacy of dexamethasone in dialysis patients with moderate II COVID-19 and found that the duration of hospital stay in the dexamethasone-treated group was significantly longer than that in the non-dexamethasone-treated group and that the 28-day survival rate was insignificant [14]. Although it is difficult to conduct a prospective RCT, further studies are required to determine whether patients on dialysis therapy have a better prognosis by treating with steroids. Regarding the type of steroid used, most of the facilities used dexamethasone, followed by methylprednisolone (Fig. 3b). The reasons for these differences include that dosage adjustment is easier with methylprednisolone due to differences in body weight between patients, that methylprednisolone has been reported to be more highly translocated to lung tissue than dexamethasone in animal studies [15], and that an RCT by Ranjbar et al. reported that methylprednisolone was better than dexamethasone in terms of ventilator requirements, ICU admission, length of hospital stay at day 5 and 10, and regarding 28-day all-cause mortality [16]. Currently, dexamethasone is the most used steroid in Japan, but the type of steroid also requires further investigation. In terms of the duration of steroids, many facilities reported that the duration of steroids should be longer than the usual 10 days (Fig. 3d). This may be because it takes longer to improve respiratory status and imaging findings in patients receiving dialysis therapy, as shown in Table 3. Another issue to consider is the effect of steroid pulse therapy. The results of this survey showed that steroid pulse therapy was used in 48.5% of facilities. In Europe and the USA, steroid pulse therapy is rarely used because of concerns regarding secondary infections. However, in Japan, our survey revealed that steroid pulse therapy is used more frequently in patients with worsening symptoms of moderate II or higher and in patients who require a ventilator because of its strong inflammatory response (Table 5). Edalatifard et al. reported that 250 mg/day steroid pulse therapy for 3 days resulted in a significantly higher clinical improvement and a lower mortality rate than the control group [17]. In a report from Japan, Tamura et al. reported the therapeutic effect of high-dose steroid pulse therapy (1 g of methylprednisolone (mPSL) for 3 days) [18]. Furthermore, Pinzon et al. reported that high-dose methylprednisolone for 3 days followed by oral prednisone for 14 days reduced recovery time and the need for intensive care compared to 6 mg dexamethasone for 7–10 days [19]. Further studies are required to determine the significance of steroid pulse therapy, the amount of steroid during steroid pulse therapy, and the incidence of secondary infections.

Another consideration is the use of tocilizumab. The survey showed that only 25.8% of the facilities had experience with tocilizumab. Tocilizumab was used for patients with moderate to severe disease and for patients on ventilators or equivalent, as was steroid pulse therapy (Table 6). Tocilizumab is an IL-6 receptor antagonist, which is expected to block inflammatory signaling and therefore control the cytokine storm in COVID-19. The COVACTA trial, which investigated the effect of tocilizumab in patients with moderate disease II (severe), failed to show a significant improvement in clinical status or reduction in 28-day mortality with tocilizumab [20]. However, the RECOVERY trial, which included SpO_2_ < 92% or oxygen administration in addition to C-reactive protein > 7.5 mg/dL, [21] and the REMAP-CAP trial in severe patients showed a significant reduction in mortality in the tocilizumab group [21]. Furthermore, Toda et al. reported the efficacy of combining tocilizumab and steroid pulse therapy in dialysis patients [22]. Further studies are required to determine the appropriate target patients and the timing of administration, and evaluate the possibility of the combination therapy, particularly in critically ill patients. However, baricitinib is contraindicated in patients receiving dialysis therapy and has not been administered, except in a few centers.

The proper choices of dialysis membranes and anticoagulants were also controversial and included in the questionnaire. Nafamostat was currently being applied in COVID-19 patients as an anticoagulant in some facilities (Table 7). Nafamostat is thought to inhibit the entry of SARS-CoV-2 into human epithelial cells by inhibiting serine 2, a transmembrane protease [23]. Doi et al. reported that nafamostat mesylate therapy, in combination with favipiravir, resulted in a lower mortality rate in patients with severe COVID-19 [24]. However, there have been few reports on its efficacy. In terms of dialysis membranes, a few facilities used PMMA membranes [25], which are known to remove cytokines (Table 7). A case series from Japan [26] described their efficacy as a cytokine adsorbent membrane, but the evidence was limited. PMX-DHPs are a therapeutic option for the selective adsorption of endotoxins and are expected to adsorb a variety of endogenous cytokines, which were used at only two facilities in this survey. Katagiri et al. used PMX-DHPs with COVID-19 requiring oxygen supply and reported that the early use of PMX columns may reduce local inflammation of the lung and prevent the subsequent demand for ventilation [27]. However, there are insufficient data on dialysis membranes and anticoagulants; hence, further research is required.

The use of ECMO in patients receiving dialysis therapy in Japan is quite limited with only ten centers. Complications of severe chronic organ failure are known to have a poor prognosis and are considered indications for exclusion.

There are issues regarding whether the discharge criteria defined by the MHLW can also applied to patients receiving dialysis therapy. Many centers use the same discharge criteria as those for patients who are not receiving dialysis therapy (10 days after onset and 72 h after symptom remission). However, our data showed that patients are still required to have negative or high Cycle threshold values in PCR tests and negative quantitative antigen tests at discharge before they return to dialysis facilities (Table 10). This may be affected by the fact that the criteria for discharge from hospital have changed by various recommendations from the beginning of the epidemic to the time of the questionnaire. In Japan, the discharge criteria also indicates that immunocompromised patients should be discussed by an infection specialist, which suggests that the responsibility for what is not known hangs on the frontline.

Finally, a question regarding DNAR in COVID-19 patients with dialysis therapy and high mortality was also included. More than half of the facilities had discussions with patients and their families about DNAR on admission, considering situations of COVID-19, where symptoms deteriorated rapidly even if they were mild. Therefore, advanced care planning should be promoted, especially during the COVID-19 pandemic.

As the limitation of this survey, there have been many changes in treatment and discharge criteria between the beginning of the epidemic and the time of the questionnaire, which are still being updated daily, therefore the results are only as of the time of the questionnaire. Furthermore, the timing of acquisition of treatment indications in Japan may have influenced our results. In addition, the emergence of variants has affected and will continue to affect the treatment of COVID-19. Additionally, the survey did not evaluate the efficacy of each treatment. Each facility is encouraged to select treatment options based on the latest evidence.

## Conclusion

This questionnaire survey revealed a variety of treatment practices in each facility for COVID-19 patients receiving dialysis therapy in Japan. Among our survey for Japanese dialysis facilities, antivirals were initially used in moderate I or more severe condition whereas steroids were greater used in moderate II and more severe conditions. However, in some standardized treatments such as steroid administration and antivirals, recommendations by MHLW have not necessarily followed. There was still high mortality rate in dialysis patients, therefore further evidence and innovations are required to improve the prognosis of patients with COVID-19 receiving dialysis therapy.

## Data Availability

All data generated or analyzed during this study are included in this published article.

## Abbreviations

CHDF: Continuous hemodiafiltration
DNAR: Do not attempt resuscitation
ECMO: Extracorporeal membrane oxygenation
ICU: Intensive care unit
PCR: Polymerase chain reaction
PMMA: Polymethylmethacrylate
PMX-DHP: Polymyxin B-immobilized fiber column-direct hemoperfusion
RCT: Randomized controlled trial
